# MicroRNA‐383 inhibits proliferation, migration, and invasion in hepatocellular carcinoma cells by targeting PHF8

**DOI:** 10.1002/mgg3.1272

**Published:** 2020-05-22

**Authors:** Yan Cheng, Na Liu, CaiFeng Yang, Jiong Jiang, Juhui Zhao, Gang Zhao, Fenrong Chen, Hongli Zhao, Yang Li

**Affiliations:** ^1^ Department of Digestive Diseases The Second Affiliated Hospital Medical School of Xi'an Jiaotong University Xi'an China; ^2^ Department of Otolaryngology–Head and Neck Surgery The Second Affiliated Hospital Xi'an Jiaotong University Xi'an China

**Keywords:** hepatocellular carcinoma, invasion, migration, miR‐383, PHF8, proliferation

## Abstract

**Background:**

To study the effect of microRNA‐383 (miR‐383) on cell proliferation, migration, and invasion of hepatocellular carcinoma (HCC) cells, and explore its mechanism.

**Methods:**

The expressions of miR‐383 and plant homology domain that refers to protein 8 (PHF8) were detected in tissues and cells by quantitative real‐time polymerase chain reaction (qRT‐PCR) or western blot respectively. The miR‐383 group (transfected miR‐383 mimics), miR‐con group (transfected miR‐con), si‐con group (transfected si‐con), si‐PHF8 group (transfected si‐PHF8), miR‐383 + ctrl group (cotransfected miR‐383 mimics and pcDNA‐3.1), miR‐383 + PHF8 group (cotransfected miR‐383 mimics and pcDNA‐3.1‐PHF8) were transfected into HepG2 cells by liposome method. Cell proliferation, migration and invasion were measured by 3‐(4,5‐dimethyl‐2‐thiazolyl)‐2,5‐diphenyl‐2‐H‐tetrazolium bromide (MTT) or trans‐well assays respectively. The luciferase activity of each group was detected by dual luciferase reporter gene assay.

**Results:**

Compared with normal adjacent tissues, the expression of miR‐383 was significantly down‐regulated and the expression of PHF8 was significantly up‐regulated (*p* < .05). Compared with normal hepatocellular cell LO2, the expression of miR‐383 was significantly reduced (*p* < .05) in HCC cells. Moreover, overexpression of miR‐383 or silencing of PHF8 significantly inhibited the proliferation, migration, and invasion of HCC cells. In addition, PHF8 was targeted by miR‐383 and its restoration rescued the inhibitory effect of miR‐383 on cell proliferation, migration, and invasion of HCC cells.

**Conclusion:**

miR‐383 could inhibit the proliferation, migration, and invasion of HCC cells by targeting PHF8, which will provide a basis for miR‐383 targeted therapy for HCC.

## Graphical abstract

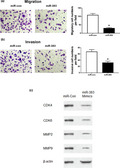

## 1 INTRODUCTION

Hepatocellular carcinoma (HCC) accounts for 90% of all cases of liver cancer, which is very common tumor malignancy with the leading causes of cancer‐related deaths worldwide (Takaya et al., 2018). Despite great advances in treatment strategies and related molecular mechanisms, the prognosis of HCC is still poor, mainly due to the higher probability of metastasis or recurrence (Bassan & Hoelzer, 2011; Collins‐Underwood & Mullighan, 2010).

Noncoding RNAs (ncRNAs), including microRNAs (miRNAs), long‐chain ncRNAs (lncRNAs), small nuclear RNAs (snRNAs), and circular RNAs (circRNAs), are major components of the human transcriptome (Weng et al., 2017). Among these, miRNAs with about 23 nucleotides in length act as important gene regulators in animals and plants (Bartel, 2009; Rupaimoole, Calin, Lopez‐Berestein, & Sood, 2016). Increasing evidence has indicated that miRNAs are often dysregulated in human malignant tumors and play key roles in a variety of biological processes, including development of HCC (Xu et al., 2018). Studies have reported that miRNAs have essential roles in the pathogenesis and diagnosis of HCC (Ji et al., 2018; Rana et al., 2018). MiR‐383 has been suggested to be lowly expressed in HCC tissues and cells (Fang et al., 2017). However, the underlying mechanism is still not fully understood.

The plant homology domain that refers to protein 8 (PHF8, also known as KDM7B or JHDM1F) is a member of histone demethylase and has recently received much attention due to its extensive expression and function as a transcriptional coactivator (Fortschegger et al., 2010; Wang et al., 2016). PHF8 binds to about one third of the promoter sites of human genes and activates their expressions, including H3K9me1/2, H3K27me2, and H4K20me1 (Qi et al., 2010; Zhu et al., 2010). These findings suggest that abnormal expression of PHF8 may be related to genetic and environmental diseases, such as human cancers. Up‐regulation of PHF8 is a key factor in the regulation of malignant progression and metastasis of multiple tumors. Studies have shown that PHF8 contributes to DNA damage protection, anti‐apoptosis, activation of cell cycle and epithelial‐to‐mesenchymal transition (EMT; Shao et al., 2017; Shen, Pan, & Zhao, 2014). Intriguingly, bioinformatics analysis provides the potential binding sites of miR‐383 and PHF8. However, there is no direct evidence in support of the interaction between miR‐383 and PHF8 in HCC.

This study will detect the expression of miR‐383 and PHF8 in HCC tissues and cells. Moreover, we will observe the effects of miR‐383 and PHF8 on proliferation, migration, and invasion of HCC cells, and reveal the potential mechanism. This may provide a new target for targeted therapy of HCC.

## 2 MATERIALS AND METHODS

### 2.1 Patients and tissues

A total of 40 patients who were diagnosed with HCC and received tumor resection in Second Affiliated Hospital of Medical School of Xi’an Jiaotong University from September 2017 to August 2019. Patients with HCC who underwent surgical operation, chemotherapy, or radiotherapy before serum collection were excluded. Presurgical blood from HCC patients and healthy subjects were sampled and stored in EDTA‐vacuum tubes. HCC tissues and adjacent nontumor tissues were obtained during surgical resection. Clinical information of all samples was retrieved from medical information system of the hospital. All subjects have signed informed consent and this study is approved by the ethic committee of the hospital. The demographic and clinical characteristics of recruited patients were summarized in Table 1.

**TABLE 1:** Demographic and clinical characteristics of 40 recruited HCC patients

Characteristics	Number of patients (%)
Age (years)	
<55	13 (32.5)
≥55	27 (67.5)
Gender	
Female	14 (35)
Male	26 (65)
Histological type	
Fibrolamellar carcinoma	2 (5)
Hepatocellular carcinoma	28 (70)
Hepatocholangiocarcinoma (mixed)	10 (25)
Histologic grade	
G1‐G2	21 (52.5)
G3‐G4	19 (47.5)
Stage	
I‐II	27 (67.5)
III‐IV	13 (32.5)
T classification	
T1‐T2	28 (70)
T3‐T4	12 (30)
N classification	
N0	33 (82.5)
N1	7 (17.5)
M classification	
M0	36 (90)
M1	4 (10)

### 2.2 Materials

Tissue specimens were collected from 43 cases of HCC specimens surgically resected and their adjacent normal tissues from February 2018 to October 2018 in our hospital. The study was approved by the medical ethics committee of the hospital, and all patients and their families have signed informed consent; Dulbecco's Modified Eagle Medium (DMEM), 3‐(4,5‐dimethyl‐2‐thiazolyl)‐2,5‐diphenyl‐2‐H‐tetrazolium bromide (MTT), fetal bovine serum, and trypsin were purchased from GIBCO; Lipofectamine ^TM^ 2000, Trizol, and TaqMan miRNA reverse transcription kit were purchased from Thermo Fisher; polyvinylidene difluoride (PVDF) membrane was purchased from Roche (Basel, Switzerland); ECL luminescent solution and RIPA protein lysate were purchased from Beyotime Biotechnology Co., Ltd.; The luciferase reporter gene detection kit was purchased from Promega; Trans‐well chambers, Matrigel were purchased from Costar; semi‐dry film converters were purchased from BIO‐RAD.

### 2.3 Cell culture

Human normal hepatocytes LO2, HCC cell lines HepG2, SK‐Hep‐1, MHCC‐LM3, and Huh7 cells were cultured in DMEM medium containing 10% fetal bovine serum. The cells were cultured in an incubator with 5% CO_2_ at 37°C.

### 2.4 Cell transfection

MiR‐383 mimics (miR‐383), miRNA negative control (miR‐con), siRNA negative control (si‐con), siRNA against PHF8 (si‐PHF8), pcDNA 3.1 (Ctrl), and pcDNA 3.1‐PHF8 (PHF8) synthesized by Genepharma (Shanghai, China) were transfected into HepG2 cells according to the Lipofectamine ^TM^ 2000 liposome instructions. After 24 hr, transfection efficacy was detected by quantitative real‐time polymerase chain reaction (qRT‐PCR). After successful transfection, it was used for follow‐up experiments.

### 2.5 qRT‐PCR experiment

The Trizol was used to extract total RNA from tissue samples or cell samples following the manufacturer's instructions. DNaseI digests DNA that may be contaminated in RNA. The template strand complementary DNA (cDNA) was synthesized according to the specification of reverse transcription kit. The operation was carried out according to the requirements of the qRT‐PCR kit reaction system. After the reaction, the relative expression level of miR‐383 was calculated by the 2^−△△Ct^ method. Each sample was repeated three times and the average value was obtained.

### 2.6 Western blot experiment

Tissues or cells were lysed in RIPA lysis buffer for 30 min and then centrifuged at 12,000 g for 10 min. The supernatant was taken into the sterile new EP tube and interacted with 5× SDS sample buffer in boiling water for 10 min. Then proteins were separated by electrophoresis, and transferred into the PVDF membrane with a film transfer machine. The membrane was sealed by 5% skimmed milk powder for 2 hr, then incubated with anti‐I overnight at 4°C and with anti‐II at room temperature for 2 hr. The luminescent liquid was added and exposed, and the ratio of the target strip gray value to the β‐actin gray value indicates the expression of the target protein PHF8.

### 2.7 MTT experiment

Suitable amount of cells in each group were cultured in 20 µl of MTT solution (5 g/L) for 3.5 hr, then added 150 µl of DMSO into each pore to make the crystallization dissolve after discarding the supernatant. The absorbance (A) was measured at 490 nm wavelength. The Cell proliferation is directly proportional to the absorbance.

### 2.8 Trans‐well experiment

The cells in each group were inoculated into 6‐well plates with 10^6^ cell/well. When the fusion degree was 80%, serum‐free medium was replaced for overnight culture. After adjusting cell density to 10^5^ cells/ml, 100 µl of cell suspension was added to the upper chamber, and 600 µl of serum‐containing medium was added to the lower chamber, followed by cultured overnight. The chamber was removed, and the cells in the upper chamber were wiped with a cotton swab. The migrated cells attached to the lower surface of the chamber were fixed with methanol for 30 min, stained with 0.1% crystal violet for 20 min, and washed with PBS. The number of migrated cells was observed under a microscope, and five fields of view were randomly selected and averaged.

The surface of the trans‐well chamber is coated with a suitable amount of Matrigel and then operated as described above. Finally, the invasive cells attached to the lower surface of the chamber were observed under microscope.

### 2.9 Dual luciferase reporter gene assay

HepG2 cells were cotransfected with luciferase reporter vectors (psiCHECK2‐PHF8‐WT, psiCHECK2‐PHF8‐MUT) and miR‐383 or miR‐NC by liposome method according to the manufacturer's protocols. After 4 hr of culture, fresh medium was replaced and then continued to be cultured for 48 hr. The luciferase activity was measured according to the instructions of the dual luciferase reporter gene assay kit. The results showed that the binding intensity of miR‐383 to PHF8 was reflected by the ratio of the luminescence intensity of sea cucumber luciferase to firefly luciferase.

### 2.10 Animal study

C57BL/6J mice were purchased from the Model Animal Research Center of Nanjing University (Nanjing, Jiangsu, China). A total of 4 × 10^7^ HepG2 cells of an early passage (less than 5) were subcutaneously injected into the lower flank of 8‐weeks old female C57BL/6J mice. After four weeks, tumors were surgically excised and pieces (~0.1 mm) of the first established HepG2 tumor were further engrafted using a Precision Trochar (Innovative Research of America, Sarasota, Florida, USA) under sterile conditions. This procedure was repeated for establishing a syngeneic HepG2 HCC mouse model for further experiments. Mice at the age of 12 weeks with a fully active immune system were used for all the experiments conducted in this study. The sizes of tumors (length × width) were measured at the indicated time points, and tumors were obtained at 4 weeks after injection. All animal experiments were approved by the University Committee on Use and Care of Animals of Binzhou City Center Hospital.

### 2.11 Statistical analysis

All data were expressed as mean ± standard deviation (x ± s) from three independent experiments by using SPSS 21.0 software (SPSS Inc.). Data between groups were compared by one‐way analysis of variance. Pairwise comparisons were performed using SNK‐*q* test. *p* < .05 was considered statistically significant.

## 3 RESULTS

### 3.1 Correlation between clinical parameter of HCC patients and miR‐383 and PHF8 expression in HCC tissues

The miR‐383 and PHF8 expression levels of HCC patients were measured by RT‐PCR, and analyzed together with individual clinical characteristics. No significant difference of miR‐383 or PHF8 level were observed between male and female patients (Table 2). Age or histological type‐related difference in miR‐383 and PHF8 were found. Notably, patients with histologic grade G1 or G2 have higher average miR‐383 expression in HCC tissues than the average of G3 and G4 patients (0.612 vs. 0.321, *p* = .00318), and the PHF8 expression display inverse correlation (1.58 vs. 3.67, *p* = .00427). TNM classification displayed substantial correlation with miR‐383 level and inverse correlation with PHF8 in HCC tissues.

**TABLE 2:** miR‐383 and PHF8 level in HCC tissues of HCC patients

Characteristics	miR‐383 level	*p*‐Value	PHF8 level	*p*‐Value
Age (years)				
<55	0.418	0.485	3.23	0.627
≥55	0.432		3.18	
Gender				
Female	0.431	0.534	3.45	0.256
Male	0.424		3.27	
Histological type				
Fibrolamellar carcinoma	0.463	0.148	3.34	0.213
Hepatocellular carcinoma	0.415		3.52	
Hepatocholangiocarcinoma (mixed)	0.437		3.18	
Histologic grade				
G1‐G2	0.612	0.00318	1.58	0.00427
G3‐G4	0.321		3.67	
Stage				
I‐II	0.535	0.00517	1.43	0.00384
III‐IV	0.217		3.96	
T classification				
T1‐T2	0.625	0.00643	1.22	0.00588
T3‐T4	0.312		3.57	
N classification				
N0	0.534	0.00542	1.34	0.00677
N1	0.267		3.98	
M classification				
M0	0.715	8.33E−4	1.45	0.00475
M1	0.234		3.89	

### 3.2 Expression of miR‐383 and PHF8 in HCC

**FIGURE 1:**
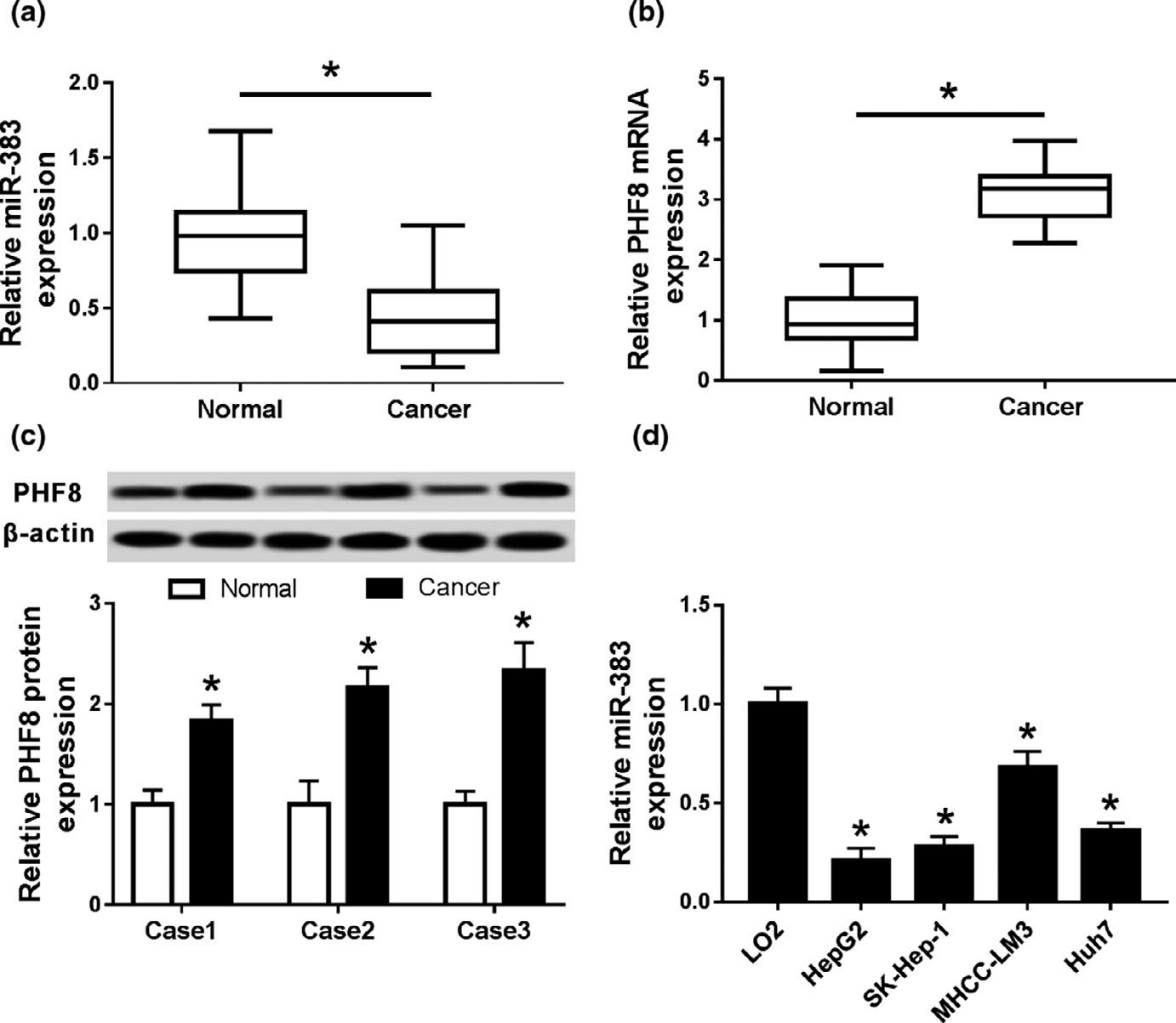
Expressions of miR‐383 and PHF8 in HCC. (a) The expression of miR‐383 in HCC and normal tissues by qRT‐PCR; (b) PHF8 mRNA expression in HCC and normal tissues by qRT‐PCR; (c) PHF8 protein expression in HCC and normal tissues by western blot; (d) miR‐383 expression in HCC cells by qRT‐PCR; *p < .05

To investigate the potential roles of miR‐383 and PHF8 in HCC, their expression levels were measured in HCC tissues and cells. Compared with that in normal tissues, the expression level of miR‐383 was significantly decreased in HCC tissues (*p* < .05) (Figure. 1a). However, the expression of PHF8 was significantly increased in HCC tissues at mRNA and protein levels (*p* < .05) (Figure. 1b,c). Moreover, the expression levels of miR‐383 were significantly decreased in HepG2, SK‐Hep‐1, MHCC‐LM3, and Huh7 compared with that in human normal liver cell LO2 (*p* < .05; Figure. 1d). These results indicated that miR‐383 and PHF8 were ectopic in HCC.

### 3.3 Overexpression of miR‐383 inhibits proliferation of HCC cells

**FIGURE 2:**
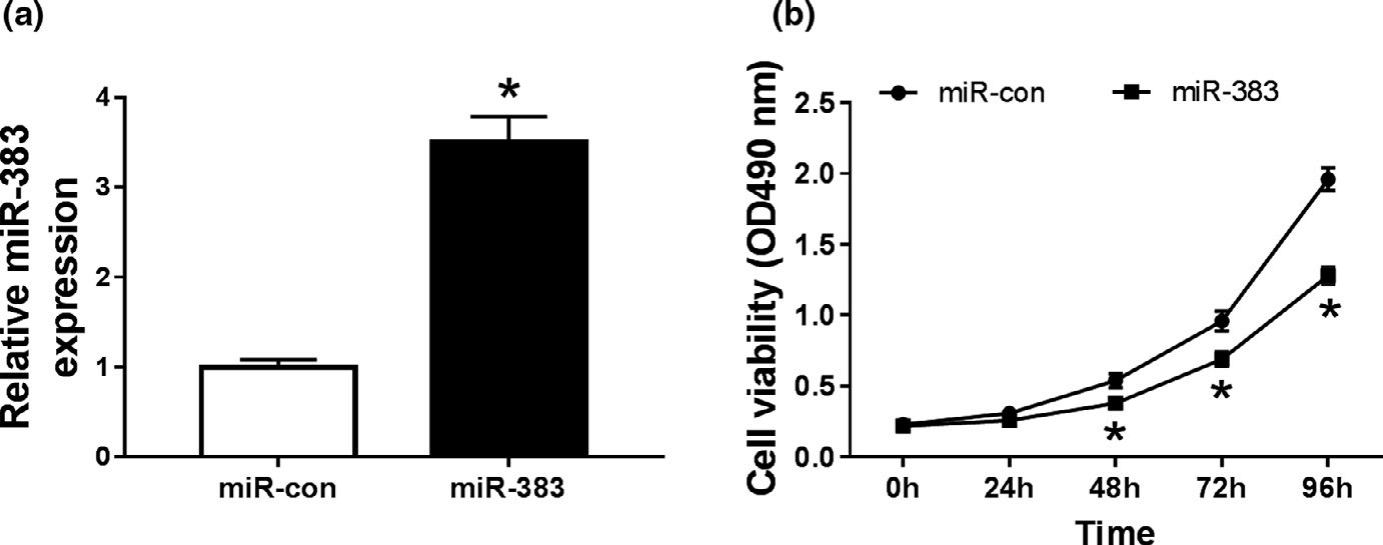
Overexpression of miR‐383 inhibits proliferation of HCC cells. (a) The expression of miR‐383 in HepG2 cells transfected with miR‐383 or miR‐con by qRT‐PCR; (b) Cell proliferation in HepG2 cells transfected with miR‐383 or miR‐con by MTT; *p < .05.

To explore the effect of miR‐383 on cell proliferation, HepG2 cells were transfected with miR‐383 or miR‐con. As a result, the abundance of miR‐383 was effectively elevated in HepG2 cells that were transfected with miR‐383 compared with that in miR‐con group (*p* < .05; Figure. 2a). Moreover, cell proliferation of each group was detected by MTT assay. Compared with the miR‐con group, the activity of cells in the miR‐383 group was significantly decreased at different time points (*p* < .05; Figure. 2b). These data suggested that overexpression of miR‐383 inhibits the proliferation of HCC cells.

### 3.4 Addition of miR‐383 inhibits migration and invasion of HCC cells

**FIGURE 3:**
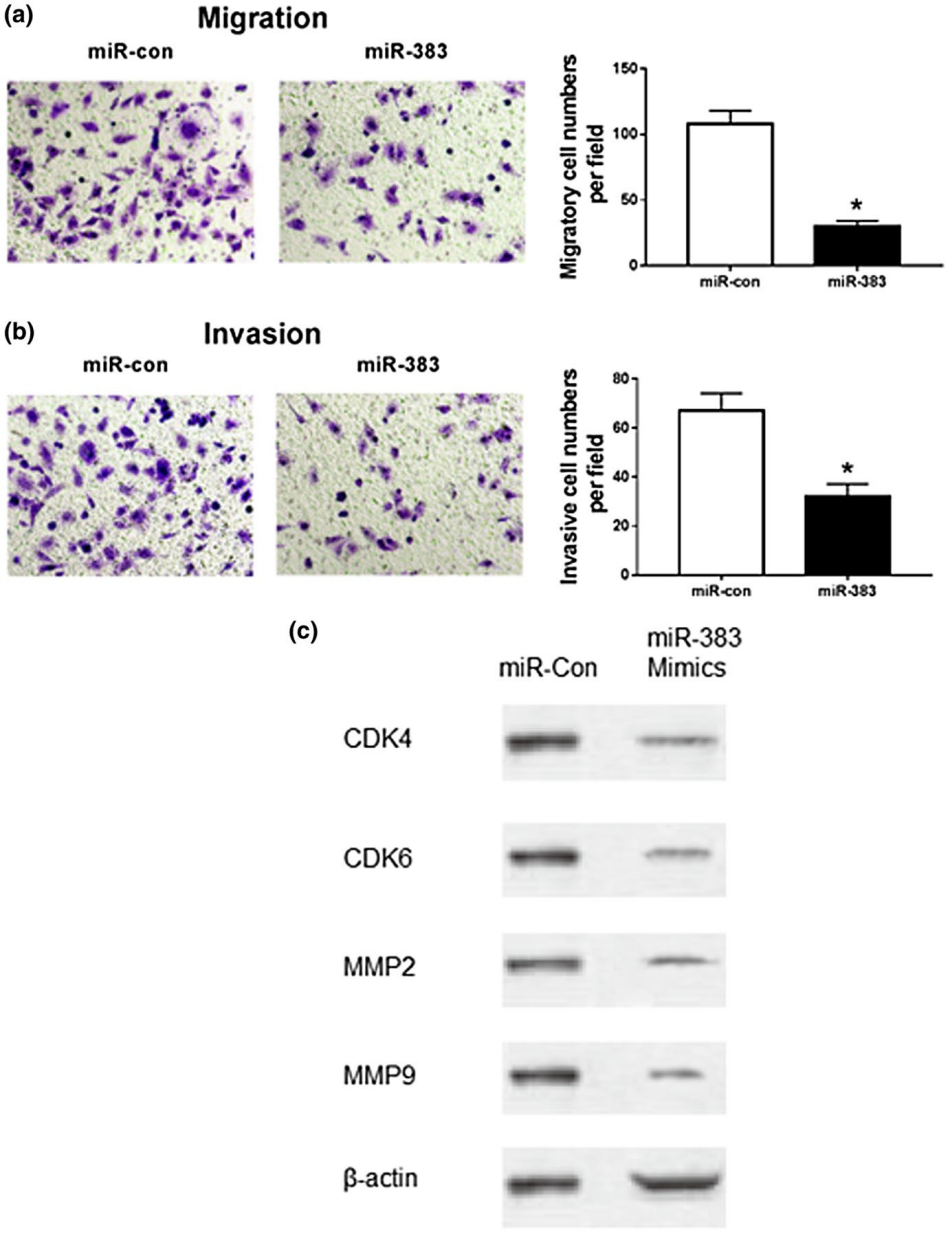
Overexpression of miR‐383 inhibits migration and invasion of HCC cells. (a) Effects of overexpression of miR‐383 on HepG2 cell migration; (b) Effects of overexpression of miR‐383 on HepG2 cell invasion; (c) Expression of proliferation‐ and invasion‐associated biomarker in cells transfected with miR‐383 mimics; *p < .05

To evaluate the effect of miR‐383 on cell migration and invasion, HepG2 cells were transfected with miR‐383 or miR‐con. Cell migration and invasion were measured by trans‐well assay. Compared with the miR‐con group, the migrated numbers of HepG2 cells were significantly reduced in miR‐383 group (*p* < .05; Figure. 3a). Similarly, overexpression of miR‐383 led to a great loss of invasive ability of HepG2 cells (*p* < .05; Figure. 3b). These results showed that overexpression of miR‐383 inhibited cell migration and invasion in HCC cells. Furthermore, western blot of proliferation related protein, CDK4 and CDK6, and migration related proteins, MMP2 and MMP9, displayed reduced expression in HepG2 cells transfected with miR‐383 mimics (Figure 3c), verifying the function of miR‐383 in proliferation and migration of HepG2 cells at molecular level.

### 3.5 Silencing PHF8 inhibits proliferation, invasion, and migration of HCC cells

**FIGURE 4:**
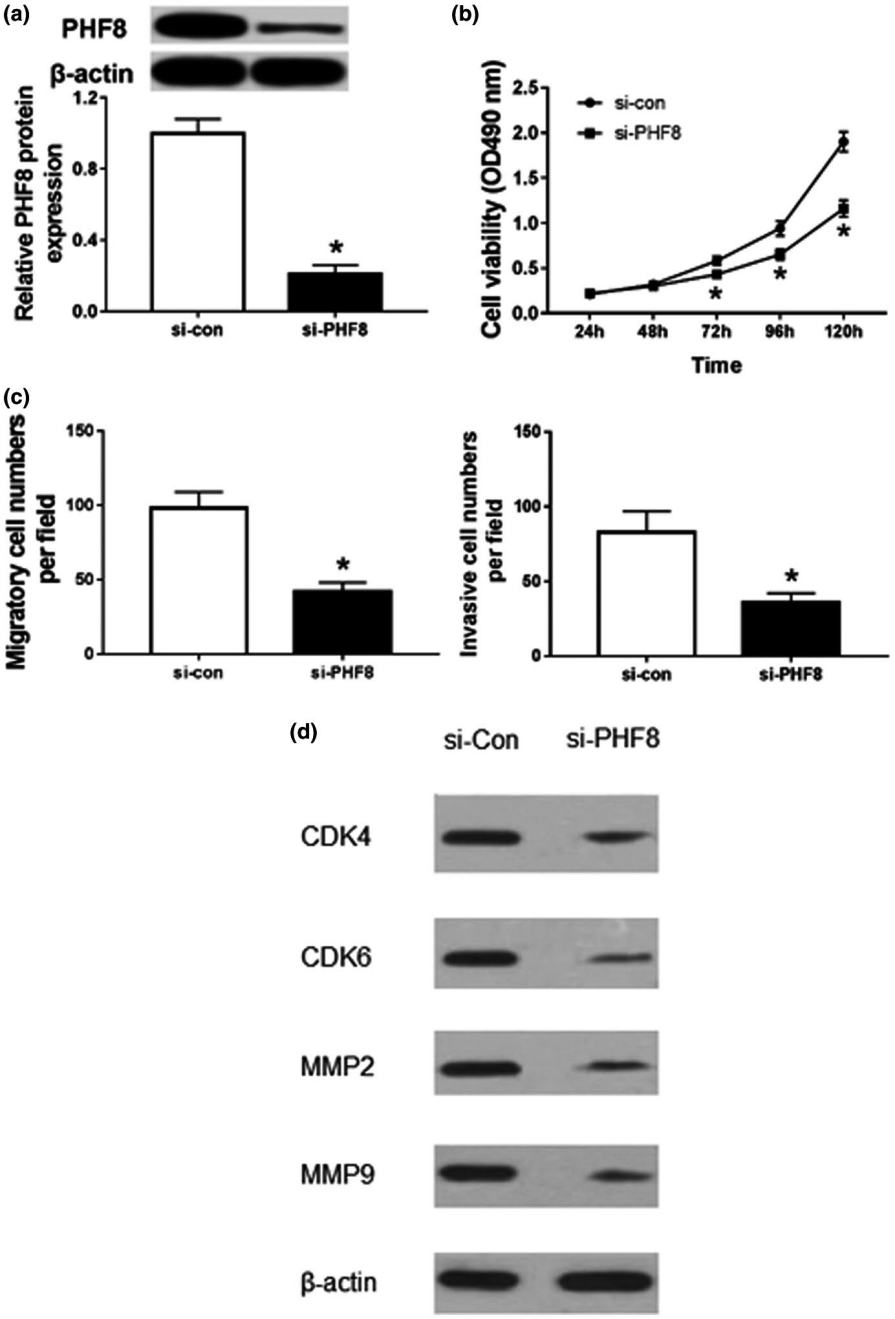
Knockdown of PHF8 suppresses proliferation, migration, and invasion of HCC cells. (a) The expression of PHF8 protein HepG2 cells transfected with si‐PHF8 or si‐con by western blot; (b) Effects of silencing of PHF8 on the activity of HepG2 cells by MTT; (c) Effects of inhibition of PHF8 on the migration and invasion of HepG2 cells by trans‐well assay; (d) Expression of proliferation‐ and invasion‐associated biomarker in PHF knocked‐down cells; *p<.05

To analyze the role of PHF8 in HCC progression, HepG2 cells were transfected with si‐PHF8 or si‐con. Compared with the si‐con group, the protein expression of PHF8 was significantly decreased in the si‐PHF8 group (*p* < .05; Figure. 4a). Moreover, knockdown of PHF8 significantly reduced cell viability (*p* < .05; Figure. 4b). In addition, cell migration and invasion were also significantly decreased in HepG2 cells transfected with si‐PHF8 compared with that in si‐con group (*p* < .05; Figure. 4c). At molecular level, the decreased expression of CDK4, CDK6, MMP2, and MMP9 in HepG2 cells transfected with si‐PHF8 evidenced that si‐PHF8 compromise the proliferative and migration capacity of HepG2 cells (Figure 4d). These findings revealed that silencing of PHF8 inhibits the proliferation, invasion, and migration of HCC cells.

### 3.6 MiR‐383 targets PHF8

**FIGURE 5:**
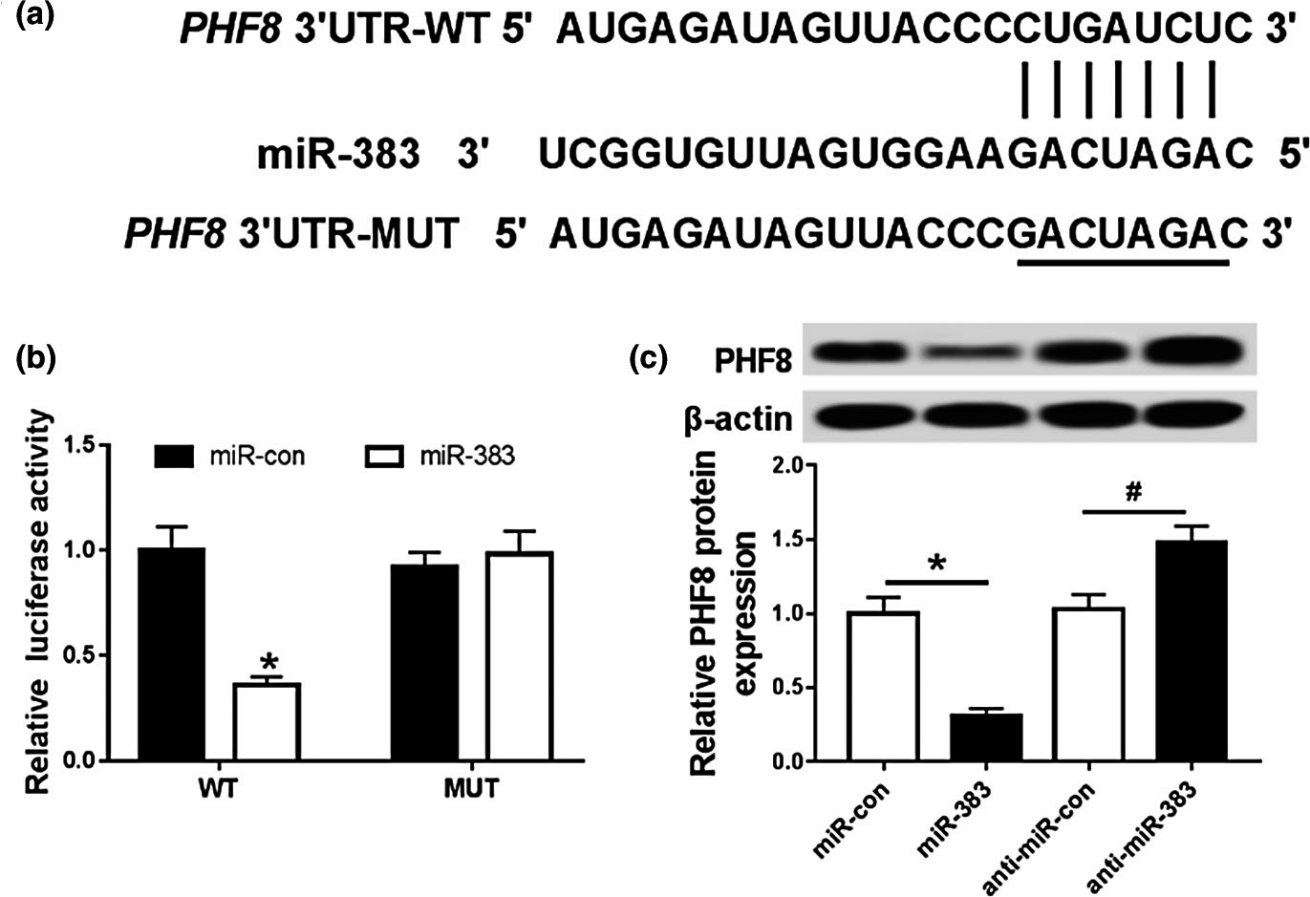
miR‐383 targets PHF8. (a) Complementary sequence of miR‐383 and PHF8; (b) Effects of miR‐383 on luciferase activity of HepG2 cells; (c) Effects of miR‐383 on expression of PHF8 protein in HepG2 cells; compared with miR‐con group, *p < .05, and compared with anti‐miR‐con group, ^#^ *p* < .05

Seeing the functions of miR‐383 and PHF8, the interaction between them was probed in HepG2 cells. The putative complementary sequences of miR‐383 and PHF8 were predicted by TargetScan, suggesting that PHF8 might be targeted by miR‐383 (Figure. 5a). To validate the prediction, luciferase activity assay was conducted in HepG2 cells. Results showed that addition of miR‐383 resulted in obvious reduction of fluorescence intensity in HepG2 cells transfected with PHF8‐WT (*p* < .05), whereas its efficacy was lost with respect to PHF8‐MUT group (*p* > .05; Figure. 5b). Moreover, the expression of PHF8 protein was significantly decreased in the miR‐383 group, while it was significantly increased (*p* < .05) in anti‐miR‐383 group compared with their corresponding control (Figure. 5c). These data uncovered that PHF8 served as a target of miR‐383 in HCC cells.

### 3.7 Introduction of PHF8 reverses the inhibitory effect of miR‐383 on proliferation, invasion, and migration of HCC cells

**FIGURE 6:**
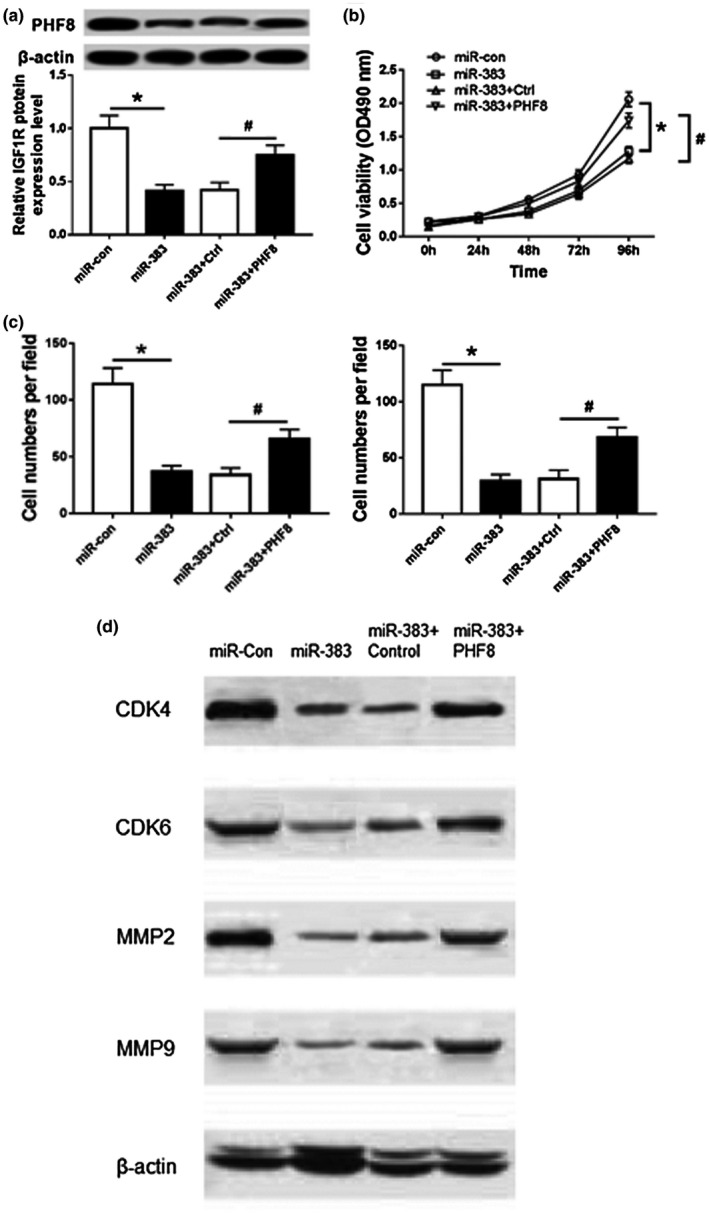
Overexpression of PHF8 reverses the inhibitory effect of miR‐383 on proliferation, invasion, and migration of HCC cells. (a) The expression of PHF8 protein in HepG2 cells cotransfected with miR‐383 and PHF8 or Ctrl; (b) Cell proliferation in HepG2 cells cotransfected with miR‐383 and PHF8 or Ctrl; (c) Cell migration and invasion in HepG2 cells cotransfected with miR‐383 and PHF8 or Ctrl; (d) Expression of proliferation‐ and invasion‐associated biomarker in cells overexpressing miR‐383 and PHF8; compared with miR‐con group, *p<.05, compared with miR‐383+Ctrl group, ^#^ *p*<.05

To explore whether PHF8 was required for miR‐383‐mediated inhibition of HCC progression, HepG2 cells were cotransfected with miR‐383 and PHF8 or Ctrl. As a result, the expression of PHF8 protein was significantly elevated in miR‐383 + PHF8 group compared with that in miR‐383 + Ctrl group (*p* < .05; Figure. 6a). Moreover, cell proliferation, migration, and invasion were measured in transfected HepG2 cells. Results showed that restoration of PHF8 reversed miR‐383‐mediated inhibition of cell proliferation in HepG2 cells (*p* < .05; Figure. 6b). Additionally, introduction of PHF8 attenuated the inhibitory effect of miR‐383 on the abilities of migration and invasion in HepG2 cells (*p* < .05; Figure. 6c). Proliferation related proteins, CDK4 and CDK6, and migration related proteins, MMP2 and MMP9, displayed reduced expression in cells transfected with miR‐383 mimics, and ectopic expression of PHF8 alleviated this inhibitory effect (Figure 6d).These results revealed that restoration of PHF8 reverses the inhibitory effect of miR‐383 on proliferation, invasion, and migration of HCC cells.

### 3.8 Murine model xenografted with HepG2 tumor transfected with miR‐383 mimics and pcDNA 3.1‐PHF8 have differentiated tumor growth and apoptosis rates

**FIGURE 7:**
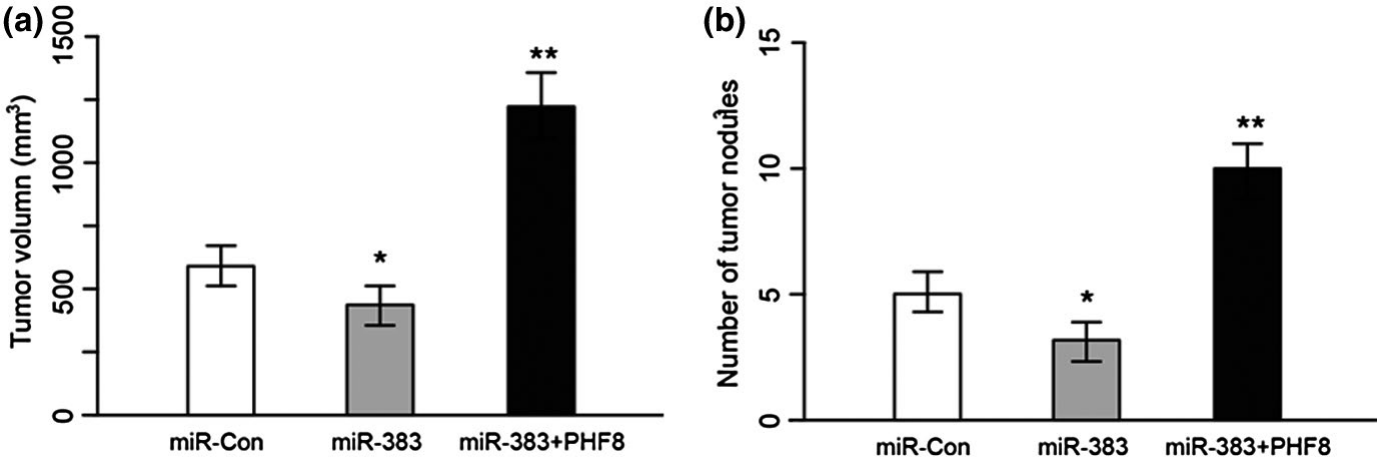
Tumor growth and apoptosis in murine model xenografted with HepG2 tumor transfected with miR‐383 mimics and pcDNA 3.1‐PHF8. (a) Tumor growth of mice xenografted with HEpG2 tumor transfected with miR‐383 mimics (*N* = 6), pcDNA 3.1‐PHF8 (*N* = 6), and contransfected of miR‐383 mimics and pcDNA 3.1‐PHF8 (*N* = 6). (b) The number of metastatic nodules in mice xenografted with HEpG2 tumor transfected with miR‐383 mimics (*N* = 6), pcDNA 3.1‐PHF8 (*N* = 6), and contransfected of miR‐383 mimics and pcDNA 3.1‐PHF8 (*N* = 6)

Xenograft of HepG2 tumor transfected with miR‐383 mimics significantly decreased the growth of HepG2 tumors compared with those transfected with miR‐con, and ectopic overexpression of PHF8 in HepG2 tumors alleviate the inhibition of miR‐383 mimics (Figure 7a). Consistently, the number of metastatic nodules observed in xenograft of HepG2 tumors transfected with miR‐383 mimics are significantly less than control, and PHF8 overexpression increased the number of nodules, indicating enhanced metastatic capacity (Figure 7b).

## 4 DISCUSSION

miRNAs, as carcinogene or tumor suppressors, can affect the malignant phenotype of tumors by participating in the development and transcriptional regulation network of tumors (Di Leva, Garofalo, & Croce, 2014). There is a one‐to‐many or many‐to‐one regulatory relationship between transcription factors and miRNAs, which increases the complexity of miRNA regulation and affects the biological characteristics of tumors (Mishra, Yadav, & Rani, 2016). MiR‐383 is lowly expressed in a variety of tumors and regulates progression of cancers by targeting multiple genes. Fang et al. (2017) found that miR‐383 is lowly expressed in HCC and its overexpression can significantly inhibit proliferation, invasion, and glycolysis of HCC cells via regulating lactate dehydrogenase A (LDHA). Moreover, Chen et al. (2016) detected the expression of miR‐383 in HCC tissues and cells. It was found that the expression of miR‐383 was low and overexpression of miR‐383 could inhibit the proliferation of HCC cells in vitro by targeting a proliferation‐inducing ligand (APRIL). In this study, qRT‐PCR was used to detect the expression of miR‐383 in HCC tissues and cells. Here, we found that miR‐383 was down‐regulated in HCC, and overexpression of miR‐383 significantly inhibited the proliferation of HCC cells, which is also consistent with previous studies. Furthermore, trans‐well assay was used to detect the migration and invasion of HCC cells. It was found that overexpression of miR‐383 could significantly inhibit the migration and invasion of HCC cells. Furthermore, we validated that PHF8 was a direct target of miR‐383 and its expression was negatively regulated by miR‐383 in HCC cells.

PHF8, a member of histone demethylase family, has been reported to be significantly up‐regulated in many malignant tumors, such as colorectal cancer (Lv, Shi, Han, & Dai, 2017), gastric cancer (Li et al., 2017), lung cancer (El‐Aarag et al., 2017), and leukemia (Fu et al., 2018). The expression of PHF8 in HCC tissues was significantly higher than that in adjacent normal tissues, and was significantly related to the malignant degree and overall survival rate of HCC, suggesting that PHF8 plays an important role in the occurrence and development of hepatocellular carcinoma. Zhou et al. (2018) used qRT‐PCR and immunochemistry to verify that PHF8 was highly expressed in HCC. Additionally, CCK8, xenograft tumor model, trans‐well assay and tandem mCherry‐GFP‐LC3 fusion protein assay showed that knockdown of PHF8 significantly inhibited HCC cells growth, migration, invasion, and autophagy. Further treatment with ChIP, western blot, and PHF8 inhibitors demonstrated that PHF8 promotes EMT, metastasis, and autophagy in HCC cells. This study indicated that the expression of PHF8 was up‐regulated in HCC tissues and cells. Moreover, silencing of PHF8 could inhibit the proliferation, migration, and invasion of HCC cells, which was also in agreement with the results of Zhou's study. In‐depth studies have found that overexpression of PHF8 can reverse the inhibitory effect of miR‐383 on proliferation, migration, and invasion of HCC cells. The in vivo study of C57BL/6J mouse model xenografted with HepG2 tumors demonstrated consistent evidence that PHF8 overexpression could reverse the anti‐proliferative and anti‐metastatic effects of miR‐383 mimics.

In summary, miR‐383 can inhibit the proliferation, migration, and invasion of HCC cells by targeting PHF8, providing a new target for targeted therapy of HCC.

## CONFLICT OF INTEREST

The authors declare that they have no competing interests.

## AUTHOR CONTRIBUTIONS

Cheng and Liu conceived and designed the study. Yang, Jiang, and Zhao were responsible for the collection and analysis of the data. Chen organized data and wrote manuscripts. Zhao, Li, and Cheng revised the manuscript critically. All authors read and approved the final manuscript.

## Data Availability

Data sharing is not applicable to this article as no new data were created or analyzed in this study.
